# Postoperative acute multiple organ failure after hepatectomy in a Nigerian male with sickle cell trait: a case report

**DOI:** 10.1186/s40792-020-01102-6

**Published:** 2021-01-13

**Authors:** Toshimitsu Iwasaki, Satoshi Nara, Yuuki Nishimura, Hiroki Ueda, Yoji Kishi, Minoru Esaki, Kazuaki Shimada, Nobuyoshi Hiraoka

**Affiliations:** 1 Department of Hepatobiliary and Pancreatic Surgery, National Cancer Center Hospital, 5-1-1 Tsukiji, Chuo-ku, Tokyo, 104-0045 Japan; 2 Division of Pathology and Clinical Laboratories, National Cancer Center Hospital, Tokyo, Japan

**Keywords:** Sickle cell disease, Sickle cell trait, Sickle cell crisis, Vaso-occlusive crisis, Multiple organ failure, Liver failure, Hepatectomy

## Abstract

**Background:**

Sickle cell disease (SCD) is a monogenic disease characterized by sickle hemoglobin (HbS). Patients homozygous for HbS experience symptoms resulting from sickled erythrocytes no later than adolescence. However, heterozygous HbS carriers, or those with the so-called sickle cell trait (SCT), may undergo surgery without their hemoglobinopathy being known.

**Case presentation:**

A 53-year-old Nigerian male with hepatitis C infection underwent radiofrequency ablation therapy for multiple hepatocellular carcinomas (HCCs) 17 months prior. Follow-up computed tomography (CT) revealed a solitary tumor (3.2 cm) in the medial section of the cirrhotic liver. The Child–Pugh score was five, and the indocyanine green retention rate at 15 min was 17.4%. The nontumorous liver of the medial section accounted for 10% of the total liver volume according to CT volumetry. With the diagnosis of recurrent HCC, left medial sectionectomy was performed under intermittent blood flow occlusion by Pringle’s maneuver. Intraoperative ultrasonography confirmed that hepatic blood flow had been preserved after hepatectomy. However, laboratory tests on postoperative day (POD) 1 revealed severe liver damage: aspartate aminotransferase 9250 IU/L, alanine aminotransferase 6120 IU/L, total bilirubin 2.8 mg/dL, and prothrombin time% 20.9%. The patient’s renal and respiratory functions also deteriorated; therefore, continuous hemodiafiltration and plasma exchange were initiated under mechanical ventilation. Whole-body contrast-enhanced CT showed no apparent ischemia of the remnant liver, but diffuse cerebral infarction was detected. Despite intensive treatments, he died of multiple organ failure on POD 20. The pathological examination of the resected specimen revealed that the intrahepatic peripheral vessels were occluded by sickled erythrocytes. Additionally, chromatographic analysis of hemoglobin detected the presence of abnormal hemoglobin, although microscopic examination of the peripheral blood erythrocytes did not show morphological abnormalities. Based on these findings, we determined that he had SCT and developed vaso-occlusive crisis involving multiple organs just after hepatectomy.

**Conclusion:**

SCD is a rare disease in eastern Asia, but its prevalence is increasing globally. Surgeons should pay increased attention to this disease, especially when performing hepatectomy under blood flow occlusion.

## Background

Sickle cell disease (SCD) is the most common monogenic disease caused by sickle hemoglobin (HbS), a variant of hemoglobin A (HbA, normal adult hemoglobin) [1]. It is estimated that over 300,000 infants are born with SCD worldwide, and approximately 100,000 individuals have SCD in the United States of America. The number of patients with SCD is expected to increase not only in high-income countries but also in low-income countries [2–4]. The incidence of SCD is high in sub-Saharan Africa (including Nigeria, Tanzania, and the Democratic Republic of the Congo), Mediterranean counties, the Middle East, and India and low in East Asian countries [5].

The most prevalent genotype of SCD is homozygous HbS (HbSS). Patients with HbSS typically show clinical manifestations such as chronic hemolytic anemia (so-called sickle cell anemia [SCA]) or repeated sickle cell crises (or vaso-occlusive crisis). The crises are due to the blocking of small blood vessels (vaso-occlusion) by sickled erythrocytes in a hypoxic state, and vaso-occlusion typically causes ischemic tissue damage and results in severe pain and/or organ failure. Patients with SCA usually experience the onset of crisis in their first or second decades of life and become aware of their SCA before reaching adulthood.

On the other hand, heterozygous carriers of HbS, who are referred to as having a sickle cell trait (SCT), are not considered to be in a disease state under normal circumstances [6]. However, under certain circumstances, such as hypoxia, hypovolemia, acidosis, hypothermia [7, 8], infection [5], or high altitude [9], individuals with SCT can unexpectedly develop sickle cell crisis, resulting in an increased risk of death. Therefore, SCT is recognized incidentally in most cases [10–12]. Here, we report a case of acute liver failure that developed after hepatectomy in a patient with previously unknown SCT.

## Case presentation

A 53-year-old Nigerian male with hepatitis C virus (HCV) infection treated with peginterferon alfa-2a and ribavirin, to which he showed a null response, presented to our hospital. Three months after the initial visit, multiple hepatocellular carcinomas (HCCs) were detected in segments 7 and 8 of the liver according to the Brisbane 2000 Nomenclature of Liver Anatomy and Resections [13], and radiofrequency ablation (RFA) was performed for each HCC. Seventeen months after RFA, a recurrence of HCC was detected in segment 4 (the medial section) by follow-up computed tomography (CT), and the patient was referred to our department.

Physical examination showed that the patient was an 87.9 kg, 168.5 cm male in good condition. The patient received medical treatment for diabetes mellitus and hypertension. He had neither a history of transfusion nor a history of SCT. The laboratory test results showed moderate liver damage, as shown in Table 1. Microscopic examination of peripheral blood erythrocytes did not show any morphological abnormalities.

**Table 1:** Preoperative laboratory data

T-Bil	0.8 mg/dL	WBC	5,200/μL
D-Bil	0.1 mg/dL	Hemoglobin	13.6 g/dL
AST	41 IU/L	PLT	150 × 10^3^/μL
ALT	50 IU/L	PT%	76%
ALP	372 IU/L	ICG-R15	17.4%
LDH	214 IU/L	Child–Pugh score	5 points
GGT	143 IU/L	AFP	6.7 ng/μL
Albumin	4.2 mg/dL	DCP	2,833 mAU/mL
HbA1c	4.2%	Anti-HCV	14.2 units

*T-Bil* total bilirubin, *D-Bil* direct bilirubin, *AST* aspartate aminotransferase, *ALT* alanine aminotransferase, *ALP* alkaline phosphatase, *LDH* lactate dehydrogenase, *GGT* gamma-glutamyltransferase, *HbA1c* hemoglobin A1C, *WBC* white blood cell count, *PLT* platelet count, *PT* prothrombin time, *ICG-R15* indocyanine green retention rate at 15 min, *AFP* alpha fetoprotein, *DCP* des-gamma-carboxy prothrombin, *anti-HCV* anti-hepatitis C virus antibody

**Fig. 1:**
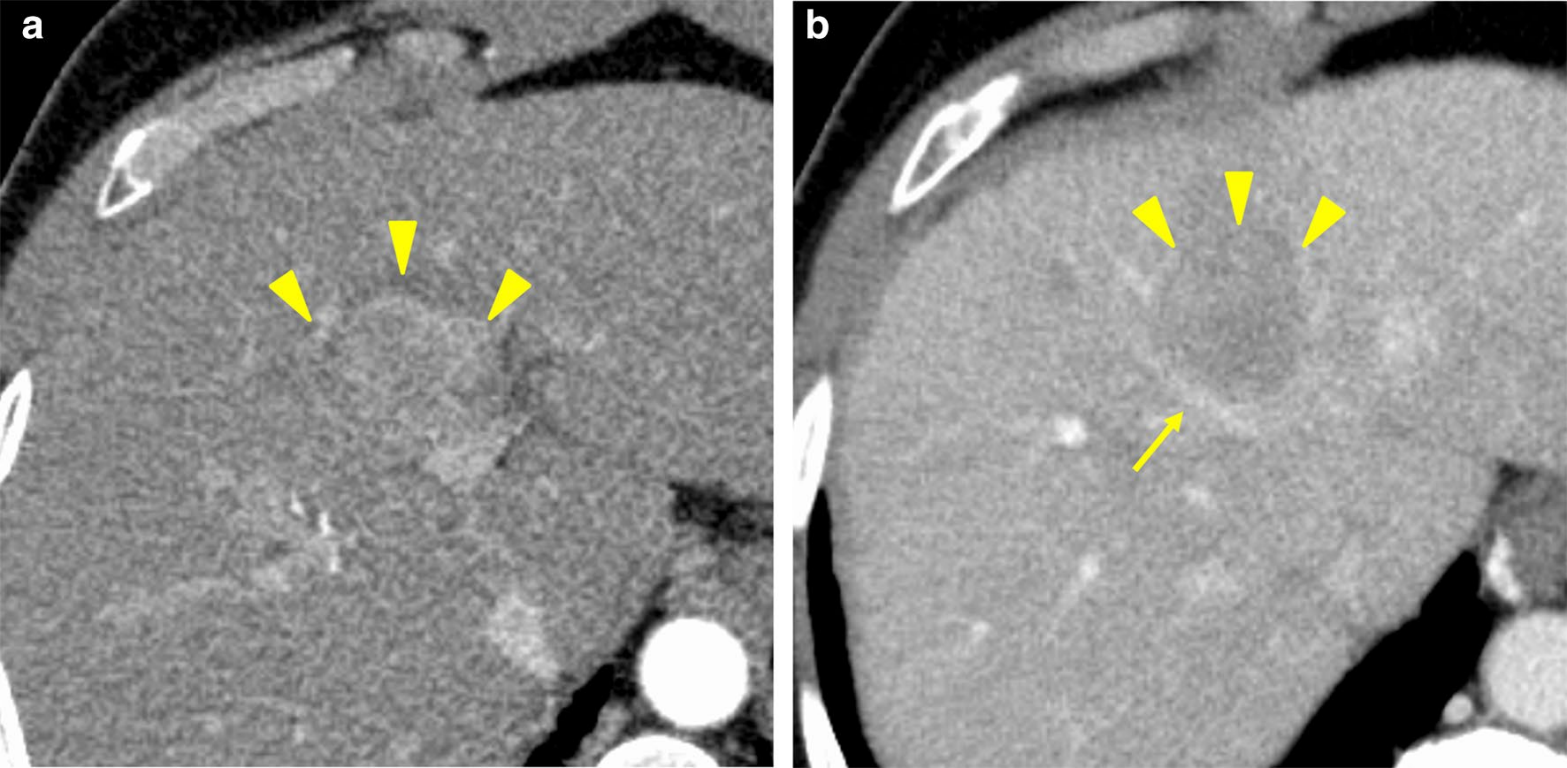
Preoperative contrast-enhanced computed tomography image of the liver. The tumor was located in the medial section of the liver (arrowheads) and showed heterogeneous enhancement in the arterial phase (**a**) and washout in the portal venous phase (**b**). The arrow indicates the middle hepatic vein

Contrast-enhanced CT showed a 4.0 × 3.4 × 3.2 cm tumor in segment 4 adjacent to the left branch of the portal vein and the middle hepatic vein (Fig. 1). An irregular liver surface; the hypertrophy of segments 1, 2 and 3; and splenomegaly all suggested the presence of cirrhosis. The normal liver parenchyma of the medial section accounted for 10% of the total liver volume according to CT volumetry.

**Fig. 2:**
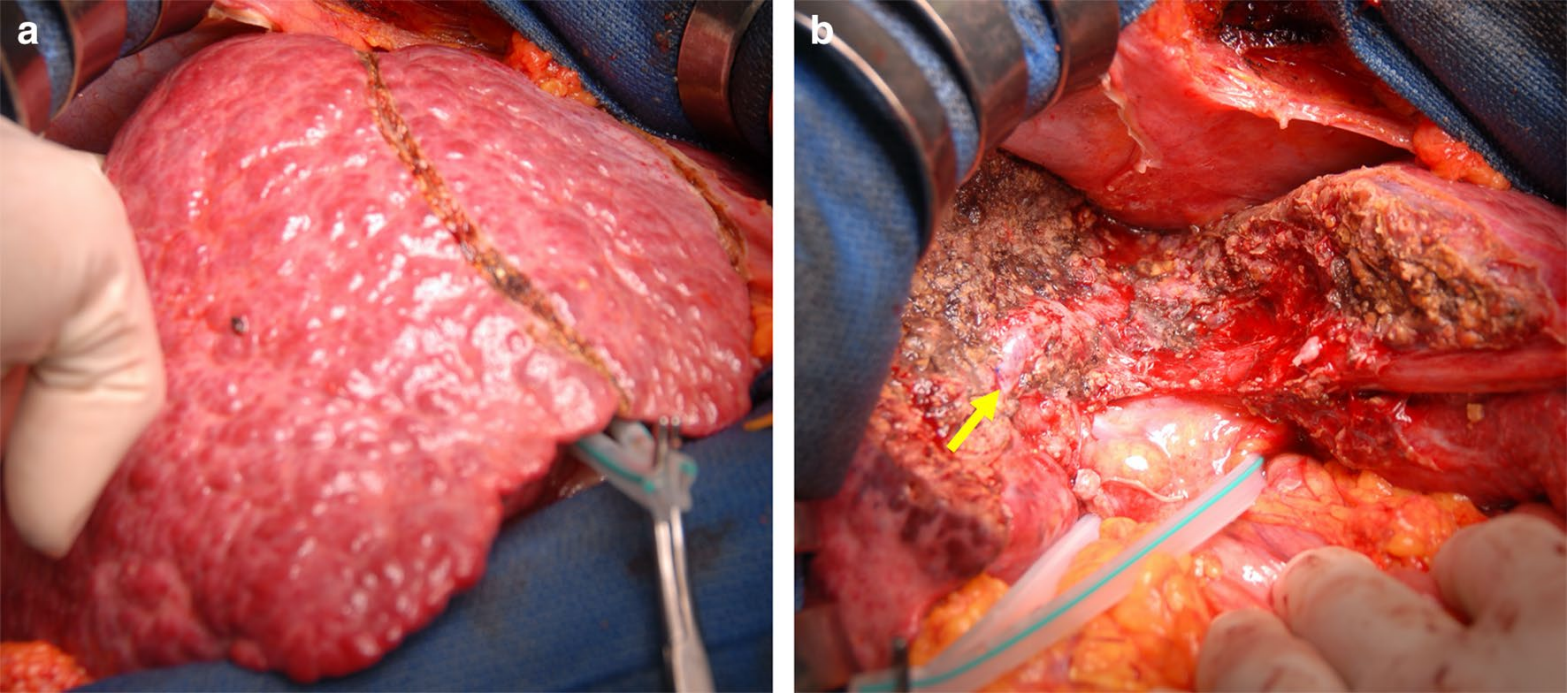
Intraoperative photographs. The nodular appearance of the liver suggested the presence of cirrhosis (**a**). After liver resection, the macroscopic appearance of the liver was normal (**b**). The arrow indicates the middle hepatic vein

With the diagnosis of solitary HCC in segment 4, left medial sectionectomy was conducted. Hepatic resection was performed with intraoperative ultrasonography (IOUS) guidance with Pringle’s maneuver (hepatic inflow occlusion time 15–30 min and reperfusion time 5 min; the total blood flow occlusion time was 165 min). Multiple IOUS examinations and inspections of the liver surface revealed no hepatic blood flow impairment during surgery (Fig. 2). At the end of the operation, laboratory data showed no conspicuous abnormalities, with total bilirubin (T-Bil) 1.6 mg/dL, aspartate aminotransferase (AST) 316 IU/L, alanine aminotransferase (ALT) 323 IU/L, hemoglobin (Hb) 11.6 g/dL, and prothrombin time (PT)% 52.5%. A drain was inserted along the resected plane of the liver. The operation time was 305 min, and the estimated blood loss was 714 mL. No blood transfusion was performed.

**Fig. 3:**
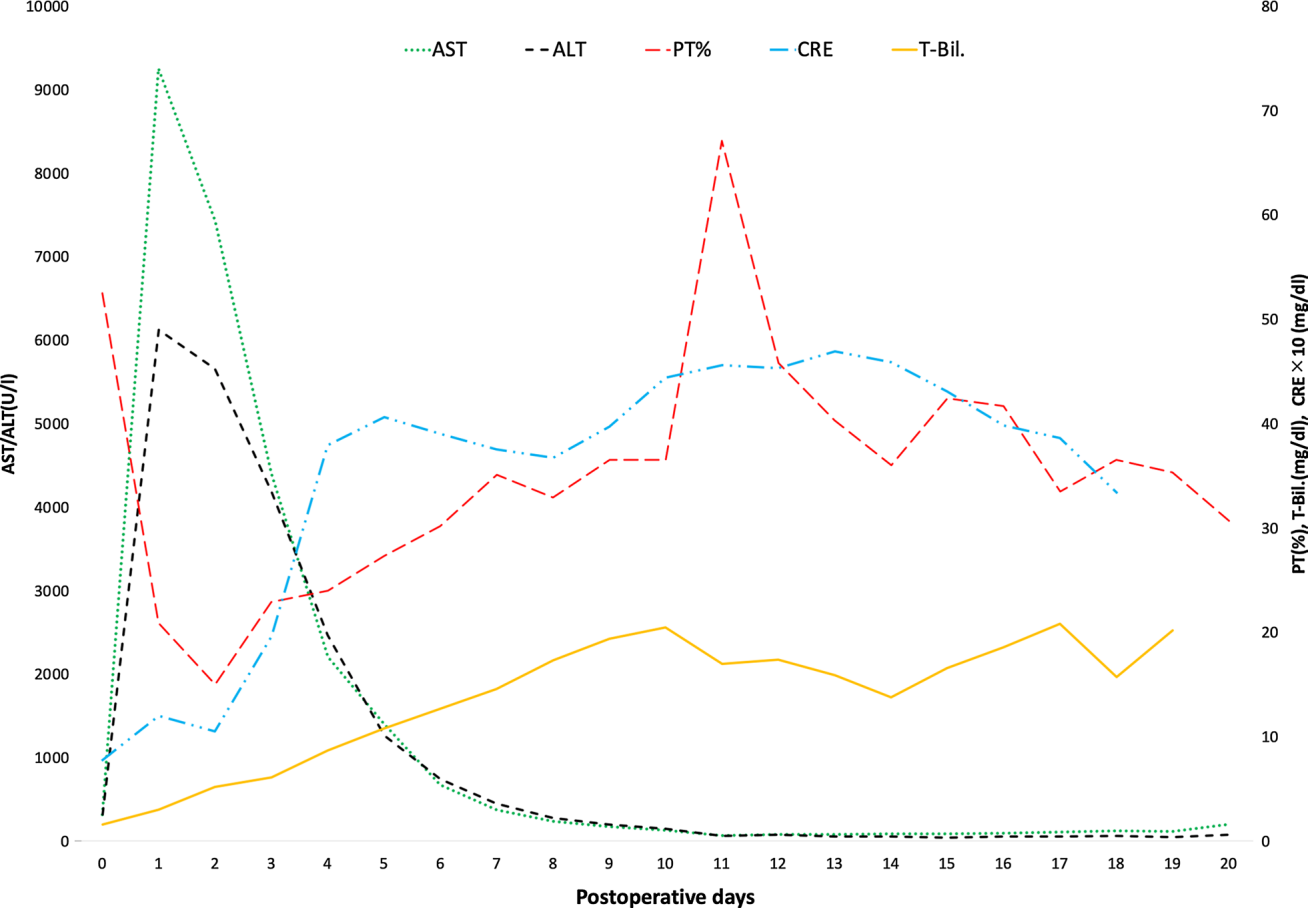
Graph shows the trends of postoperative laboratory data. AST: aspartate aminotransferase, ALT: alanine aminotransferase, PT%: prothrombin time%, T-Bil: total bilirubin, CRE: creatinine. The creatinine level was multiplied ten times and plotted on the graph

**Fig. 4:**
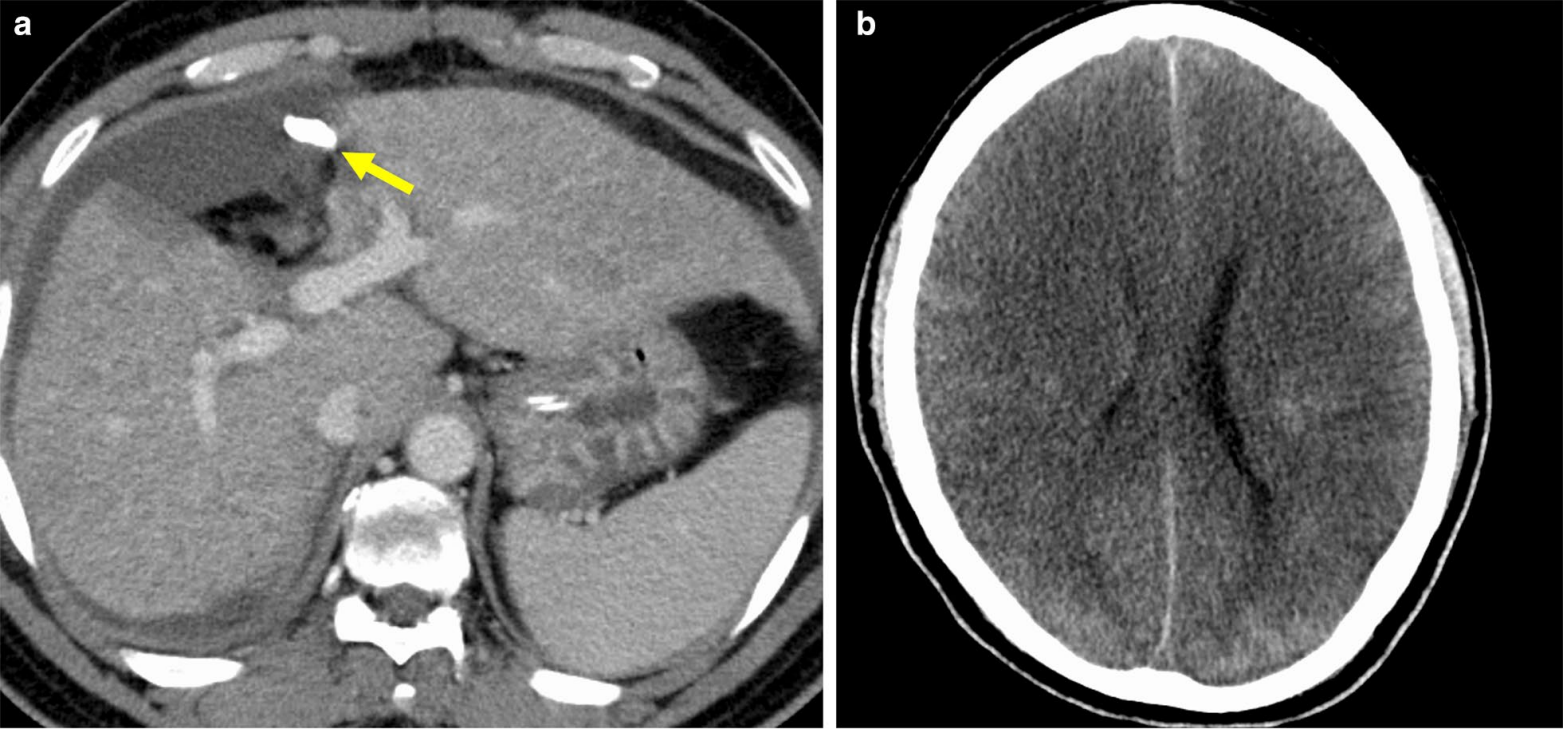
Contrast-enhanced computed tomography image performed on postoperative days 13 (**a**) and 16 (**b**). Liver parenchyma showed a slight inhomogeneous enhancement without apparent occlusion of the hepatic arteries and portal vein. The arrow indicates an abdominal drain (**a**). Diffuse cerebral edema and impaired enhancement of cerebral parenchyma were noted (**b**)

However, the laboratory tests on postoperative day (POD) 1 revealed severe liver damage and acidosis: T-Bil 2.8 mg/dL, AST 9250 IU/L, ALT 6120 IU/L, PT% 20.9%, blood pH 7.29, lactate 6.6 mmol/L, and base excess − 5.4 mmol/L. Figure 3 shows the trends of postoperative laboratory data. The bedside US did not indicate an impairment of hepatic blood perfusion. The drain output was 460 mL/15 h, with serosanguineous appearance. Because his vital signs were stable and it was difficult to identify a cause of the acute severe liver damage, we selected conservative management while performing fresh frozen plasma transfusion. The values of AST and ALT declined gradually, but the level of T-Bil continued to increase, and renal and respiratory dysfunction emerged and subsequently deteriorated; therefore, we initiated continuous hemodiafiltration and plasma exchange under mechanical ventilation. Contrast-enhanced CT showed a slightly heterogeneous enhancement of the liver parenchyma without apparent major vessel occlusion on POD 13 (Fig. 4a), and diffuse cerebral infarction on POD 16 (Fig. 4b).

**Fig. 5:**
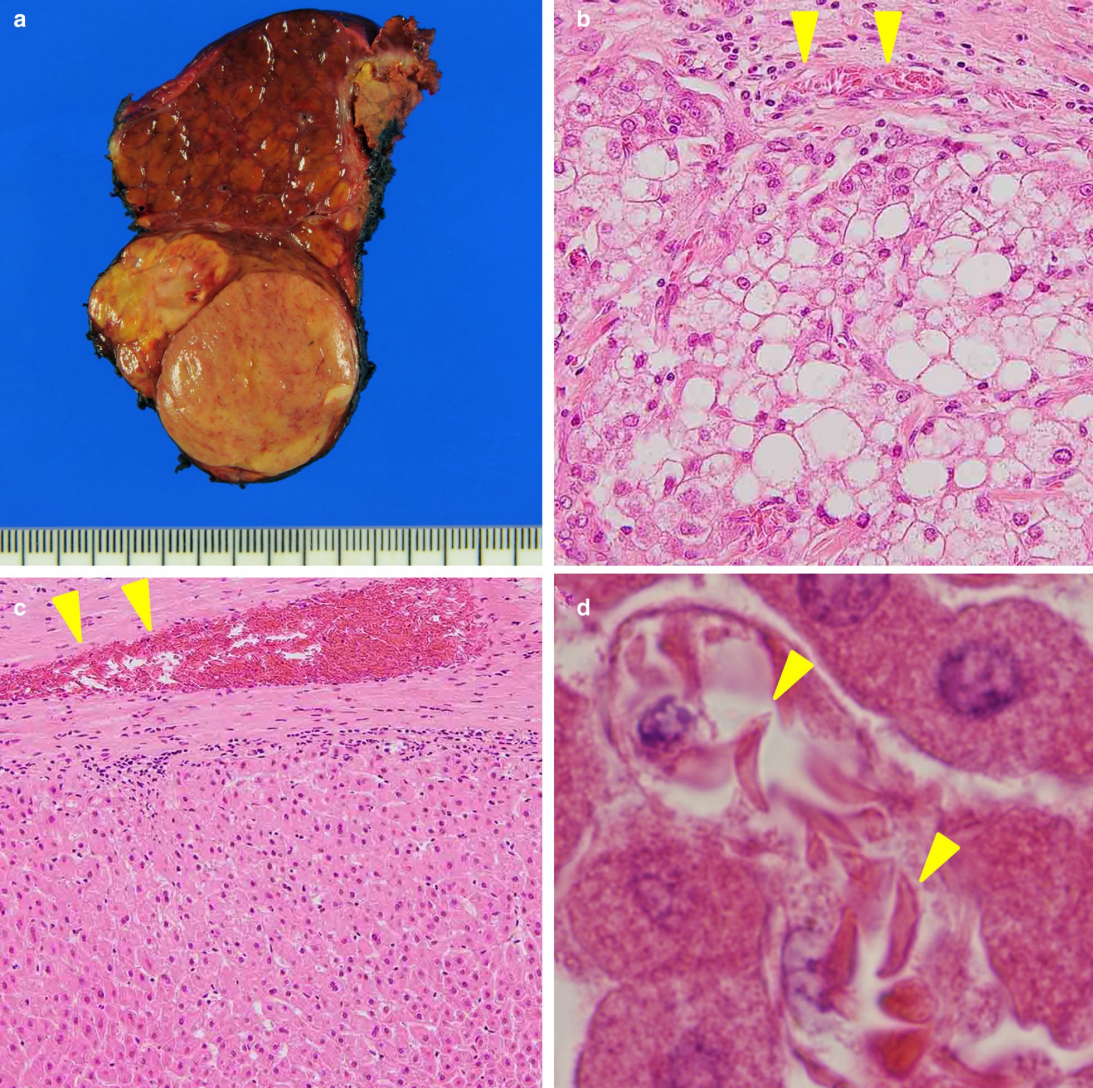
Cut surface of the resected specimen showed a single nodular hepatocellular carcinoma, 3.9 cm in size (**a**). Intrahepatic peripheral blood vessels were filled with sickled erythrocytes in both tumor (**b**) and non-tumor areas (**c**). Sickled erythrocytes were observed in the sinusoidal space (**d**)

**Fig. 6:**
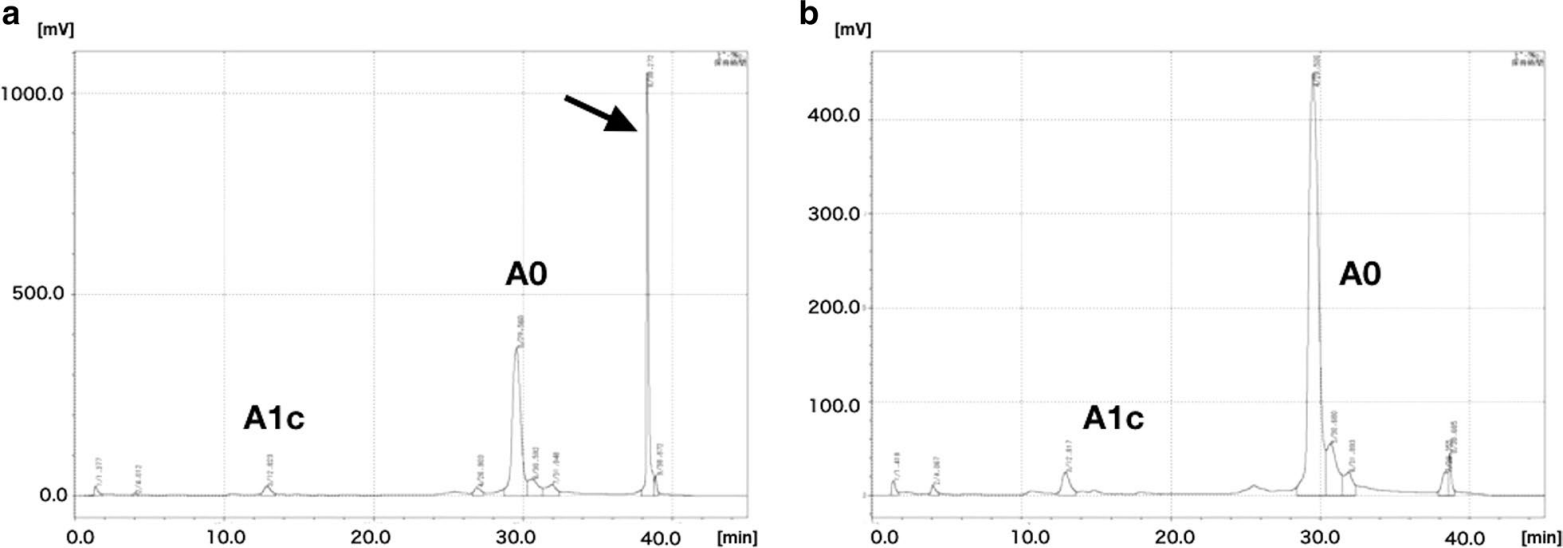
Elution curve obtained by high-performance liquid chromatography analysis of the patient (**a**) and a healthy control (**b**). An elution peak corresponding to HbS is indicated by the arrow (**a**)

Although clinico-radiological examinations could not identify the cause of acute liver damage, the pathological examination of the resected specimen revealed that the intrahepatic peripheral blood vessels were occluded by sickled erythrocytes (Fig. 5). Morphological abnormalities were not found by microscopic examination of the peripheral blood erythrocytes after surgery, but we requested an analysis of the patient’s hemoglobin by high-performance liquid chromatography (HPLC) with a suspicion of hemoglobinopathy. HPLC demonstrated an abnormal sharp peak indicating the presence of abnormal hemoglobin, which accounted for 34.4% of total hemoglobin (Fig. 6). These findings strongly suggested the presence of HbS. Despite intensive treatments, he died of multiple organ failure on POD 20. Autopsy was rejected by his bereaved family for religious reasons.

## Discussion

We speculated the pathophysiology of this patient as follows: (1) hepatectomy under intermittent hepatic blood flow occlusion resulted in a hypoxic state in the liver and caused the sickling of erythrocytes, (2) subsequent vaso-occlusion of peripheral vessels by sickled erythrocytes in the remnant liver and other organs (brain, kidney, lung, etc.) caused multiple organ failure, and (3) acute liver failure caused by impaired liver perfusion might have further contributed to the deterioration of other organ function.

Hypoxia, hypovolemia, hypothermia and acidosis are well-known risk factors leading to the sickling of erythrocytes in patients with SCD [5, 7, 8]. Such stresses cause HbS polymerization and result in erythrocyte damage and hemolytic anemia or vaso-occlusion. The clinical manifestations of vaso-occlusive crisis in patients with SCD include acute liver failure, renal failure, acute chest syndrome (respiratory failure), acute ischemic stroke, mesenteric ischemia, etc. [1, 5, 14, 15]. The crisis is thought to be caused by a variety of complications, including tissue ischemia, vascular-endothelial dysfunction, functional nitric oxide deficiency, inflammation, reperfusion injury, etc., in each organ [1, 5].

The treatment of vaso-occlusive crisis includes blood transfusion, including exchange transfusion if necessary, and extensive organ support, such as mechanical ventilation, blood purification, and plasma exchange. However, once the vaso-occlusive crisis has occurred, the prognosis of patients is very poor, with 9–33% mortality rates [16, 17]. Therefore, the prevention of crises is crucially important, especially for patients who are scheduled to undergo surgery. This includes preoperative transfusion (target hemoglobin level, 10 g/dL) and avoiding the following: hypoxia, inadequate hydration, hypothermia, and acidosis [5, 7, 8, 18].

Pringle’s maneuver is effective in decreasing bleeding during hepatectomy [19] and is routinely used during liver resection in our hospital. The standard blood flow occlusion time is 15 min in cirrhotic patients and up to 30 min in normal liver patients [20]. In this patient, the prolongation of vascular occlusion (30 min in maximum) was necessary, because the liver resection proceeded very slowly due to cirrhotic liver parenchyma and high hepatic venous pressure. Considering the intraoperative situation, total avoidance of vascular occlusion during hepatectomy may have been difficult, but shorter vascular occlusion time or selective vascular occlusion [21, 22] would have been preferable in this patient to minimize the risk of vaso-occlusion caused by liver ischemia.

Regarding hepatectomy for a patient with SCD, there is only one case report [23]. A female patient with a preoperatively known diagnosis of SCD (HbSS) developed intrahepatic cholestasis after right hepatectomy for liver metastasis of colon cancer. However, this approach was successfully managed by conservative management, including exchange transfusion. It should be noted that the presence of SCD was recognized before operation, the authors avoided the use of intermittent blood flow occlusion (Pringle’s maneuver) during hepatectomy, and meticulous postoperative management was performed to prevent SCD-associated complications.

It is essential to diagnose the presence of SCD before surgery to make adequate preparation. However, it is controversial whether preoperative screening for HbS should be performed routinely [6, 24]. Selective screening may be an option for patients from areas, where SCD is highly prevalent. However, global population movement is becoming increasingly common, making it more difficult to decide who should be tested. Thus, routine screening based on patient nationality alone is not recommended [6, 25]. Patients homozygous for HbS (HbSS) usually experience symptoms before the age of 16, and such patients are referred to a specialized team before surgery. Therefore, it is estimated that in the United Kingdom, patients with HbSS are rarely scheduled for surgery without the presence of SCD being known [25].

On the other hand, a considerable number of individuals heterozygous for HbS may be referred to surgery without the presence of SCT being known because of its relatively benign nature. It is estimated that 7.3% of blacks, 0.7% of Hispanics, and 1.6% of United States residents are heterozygous HbS carriers (SCT) [26]. Although it is considered that anesthesia for individuals with SCD is safe in ordinal surgical procedures, it is significantly important to identify a patient with SCT when performing high-risk surgery, including cardiopulmonary bypass, procedures that employ tourniquets or vascular clumping [24], because such procedures have a potential risk of causing vaso-occlusion crisis. Therefore, preoperative screening should be undertaken on a selective basis considering the patient’s national origin, medical or family history, and surgical risk (i.e., operation time and procedure) [24, 25, 27].

To date, several screening techniques for SCD are utilized depending on their advantages and limitations [28]. In Europe, HPLC, capillary electrophoresis (CE), isoelectric focusing (IEF) and tandem mass spectrometry are recommended as appropriate methods for newborn screening for SCD [29]. Among these methods, HPLC, CE, and IEF have the ability to separate and precisely quantify hemoglobin fractions [30, 31]. Therefore, these three methods are recommended as screening tests for SCT before high-risk surgeries.

In our case, the value of HbA1c (4.2%) at preoperative work-up was unexpectedly low, even considering the effect of treatment for diabetes mellitus. Recently, Lacy et al. reported that HbA1c may systematically underestimate past glycemia in black patients with SCT based on analyses of large, well-established cohorts [32]. Therefore, the unexpectedly low level of HbA1c would have been key to revealing the presence of hemoglobinopathy.

In Japan, an area with a low prevalence of SCD [33, 34], we conducted hepatectomy with Pringle’s maneuver without concern for the presence of HbS in a Nigerian patient. The delay in understanding the pathogenesis of our patient’s multiple organ failure was not a little influenced by our lack of awareness of SCD. There was only one case report of abdominal surgery in a patient with previously unknown SCT [12], where an Indian female underwent distal pancreatectomy with splenectomy for a solid pseudopapillary neoplasm of the pancreas. Microscopic examination revealed sickled erythrocytes in the blood vessels in the resected pancreatic tumor and spleen. Unlike our case, the postoperative course was uneventful. Our patient’s dismal outcome may be attributed to the acute progression, severity, and synchronicity of multiple organ failure.

Advances in surgical techniques and perioperative management have made surgery safer even for patients with SCD, especially in low-/medium-risk surgeries [18, 35]. However, we should be aware that high-risk surgeries, including neurovascular or cardiovascular surgeries that require intraoperative hypothermia [7] and hepatectomy or cancer surgeries that are associated with decreased intraoperative blood perfusion, still involve substantial risk for patients with SCD.

## Conclusion

SCD is a rare disease in eastern Asia, but its prevalence is rising globally. Surgeons should pay increased attention to this disease, especially during the perioperative period of high-risk surgery.

## Data Availability

Not applicable.

## Abbreviations

SCD: Sickle cell disease
Hb: Hemoglobin
HbS: Sickle hemoglobin
SCT: Sickle cell trait
HCC: Hepatocellular carcinoma
POD: Postoperative day
HbA: Hemoglobin A
HbSS: Homozygous HbS
SCA: Sickle cell anemia
HCV: Hepatitis C virus
RFA: Radiofrequency ablation
T-Bil: Total bilirubin
AST: Aspartate aminotransferase
ALT: Alanine aminotransferase
ALP: Alkaline phosphatase
LDH: Lactate dehydrogenase
PT: Prothrombin time
INR: International normalized ratio
CT: Computed tomography
IOUS: Intraoperative ultrasonography
HPLC: High-performance liquid chromatography
